# Endoscopic Treatment of Acute Cholelithiasis Using AXIOS Stenting and Lithotripsy: A Case Series

**DOI:** 10.7759/cureus.34643

**Published:** 2023-02-05

**Authors:** Ariana R Tagliaferri, Gabriel Melki, Yana Cavanagh

**Affiliations:** 1 Internal Medicine, St. Joseph's Regional Medical Center, Paterson, USA; 2 Medicine, St. Joseph's University Medical Center, Paterson, USA; 3 Gastroenterology, St. Joseph's Regional Medical Center, Paterson, USA

**Keywords:** gallbladder drainage, endoscopy, gastroenterology, transluminal stent, cholecystitis, cholelithiasis, lithotripsy, axios stenting

## Abstract

The management of gallbladder diseases, including acute cholecystitis and choledocholithiasis, puts a significant strain on healthcare. The first-line treatment for acute cholecystitis is cholecystectomy. Patients who have concomitant choledocholithiasis, large stones, and/or gallstone pancreatitis may also benefit from endoscopic interventions. Endoscopic treatments may also be utilized in patients who are not surgical candidates due to underlying comorbidities. Studies examining the role of endoscopic lithotripsy in concomitant cholecystitis are limited. Herein we present a case series in which an AXIOS stent (Boston Scientific, Marlborough, Massachusetts) was placed into the gallbladder for decompression and utilized to access the gallbladder lumen to perform electrohydraulic lithotripsy in two patients.

## Introduction

Approximately 20 million people in the USA are affected by gallbladder disease, with 200,000 new cases of acute cholecystitis each year [1]. Concomitant choledocholithiasis occurs in up to 20% of patients, complicating admissions for acute cholecystitis [2]. As a result, the management of gallbladder disease puts a significant strain on our healthcare system, with an annual capita exceeding 6.6 billion dollars [2]. Endoscopic therapies decrease the overall burden by reducing the overall costs and minimizing complications and hospital stays while providing definitive treatment of gallbladder disease.

The first-line treatment for acute cholecystitis is cholecystectomy; however, patients who have concomitant choledocholithiasis, large stones, and/or gallstone pancreatitis may benefit from endoscopic interventions [1, 2]. The American Society for Gastrointestinal Endoscopy (ASGE) standard of practice (SOP) guidelines recommend endoscopic retrograde cholangiopancreatography (ERCP) for the treatment of choledocholithiasis, but studies examining the role of ERCP with lithotripsy in concomitant cholecystitis are limited [2]. Often, patients who are not surgical candidates undergo emergent percutaneous biliary drainage as a bridge to definitive treatment or as part of their clinical optimization, but this comes with significant complications. Biliary decompression can also be achieved endoscopically through the use of the AXIOS stent system (Boston Scientific, Marlborough, Massachusetts), which is a dumbbell-shaped, silicone-covered stent that serves as a lumen conduit to drain solid and liquid components from pancreatic fluid collections [1, 3].

## Case presentation

Case one

A 94-year-old female with a past medical history of dementia, hypothyroidism, hypertension, and atrial fibrillation on anticoagulation, presented to the emergency department (ED) with acute epigastric pain, nausea, and vomiting. On examination, she was tachycardic and had severe epigastric tenderness without peritonitis. Laboratory studies were remarkable for acute kidney injury, unconjugated hyperbilirubinemia, elevated lipase, mixed hepatocellular-cholestatic pattern of liver injury, and leukocytosis (Table 1).

**Table 1:** Laboratory abnormalities for patient one

Laboratory abnormality	Laboratory value	Reference range
Creatinine	1.35 mg/dL	0.6-1.3 mg/dL
Total bilirubin	3.5 mg/dL	0.3-1.1 mg/dL
Lipase	7870 unit/L	11-82 unit/L
Alkaline phosphatase	313 unit/L	34-104 unit/L
Aspartate aminotransferase	213 unit/L	13-39 unit/L
Alanine aminotransferase	190 unit/L	7-52 unit/L
White blood cell count	15.7 x 10^3/mm3	4.5-11 x 10^3/mm3

**Figure 1:**
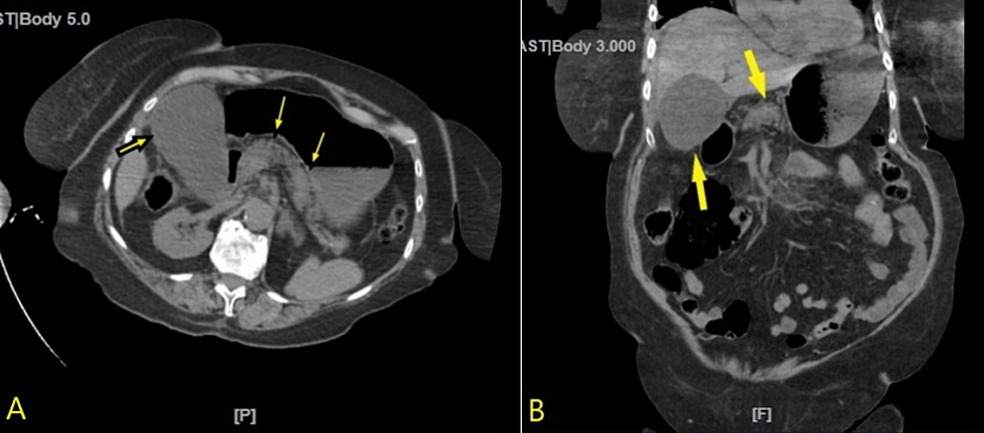
Computerized tomography of the abdomen and pelvis without intravenous or oral contrast demonstrating pancreatitis and distended gallbladder Sagittal (A) and coronal (B) imaging through the abdomen and pelvis demonstrating a markedly distended gallbladder (yellow arrows), with mild peripancreatic thick inflammatory changes suggestive of pancreatitis (yellow arrows).

A computerized tomography (CT) of the abdomen and pelvis without contrast revealed pancreatitis, duodenitis, a dilated gallbladder, and perihepatic fluid (Figure 1).

**Figure 2:**
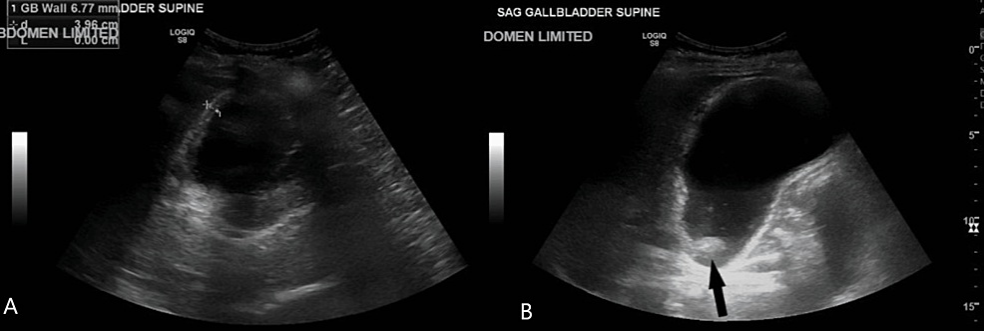
Ultrasound of the abdomen revealing cholelithiasis and acute cholecystitis There is no intra or extrahepatic biliary ductal dilation and the common duct is normal in caliber measuring 4.5 mm. The gallbladder is distended with stones (black arrow, B) and wall thickening measuring up to 6.8 mm (measurement, A). Both images (A & B) are of the gallbladder in the supine position. There is trace pericholecystic fluid and a positive sonographic Murphy's sign.

An abdominal ultrasound demonstrated an impacted gallstone within the neck of the gallbladder, thickened gallbladder wall measuring 6 mm, trace pericholecystic fluid, and a sonographic Murphy's sign (Figure 2).

The patient was diagnosed with gallstone pancreatitis and cholelithiasis with acute cholecystitis. She was treated with intravenous fluids and piperacillin-tazobactam whilst awaiting a surgical consultation. She was also evaluated by interventional radiology, who felt due to her comorbidities, leukocytosis, and elderly age, she was at high risk for percutaneous drainage. Due to her underlying comorbidities, the patient was also not a surgical candidate and subsequently underwent endoscopic cholecystogastrostomy. During endoscopic ultrasound (EUS), gallbladder sludge and two stones measuring up to 14 mm were visualized in the gallbladder. Purulent material was aspirated from the gallbladder lumen, which was dilated due to cystic duct obstruction. A cholecystogram was obtained under EUS guidance. Cholecystogastrostomy of the gastric antrum was performed using the AXIOS stent delivery system, and a 6-7-8 mm anastomotic balloon was used to dilate the internal diameter of the AXIOS stent. Sutures fixated the stent into position. She was treated with ampicillin-sulbactam for seven days and subsequently underwent an ERCP with lithotripsy upon maturation of the cholecystogastrostomy tract. During the procedure, the gallbladder was intubated via the AXIOS stent. Sludge and an 8 mm stone were found. Lithotripsy with the electrohydraulic instrument was successful in stone fragmentation and removal utilizing the endoscope. Cultures returned positive for *Enterococcus faecium*, and she was continued on ampicillin-sulbactam for another week. The patient clinically improved and was discharged to a subacute rehabilitation center. The AXIOS sent was to be removed approximately six weeks later; however, the patient suffered a pulmonary embolism while in rehab and had poor outcomes. She was ultimately transferred to a hospice center.

Case 2

A 92-year-old female with a past medical history of carotid artery stenosis, urinary incontinence, and hypertension presented to the ED with acute epigastric pain. Laboratory studies were significant for mixed hepatocellular-cholestatic liver injury, conjugated hyperbilirubinemia, and leukocytosis. Lipase was within normal limits (Table 2).

**Table 2:** Laboratory abnormalities for patient two

Laboratory abnormality	Laboratory value	Reference range
Total bilirubin	3.0 mg/dL	0.3-1.1 mg/dL
Direct bilirubin	1.8 mg/dL	0-0.2 mg/dL
Lipase	14 unit/L	11-82 unit/L
Alkaline phosphatase	105 unit/L	34-104 unit/L
Aspartate aminotransferase	168 unit/L	13-39 unit/L
Alanine aminotransferase	84 unit/L	7-52 unit/L
White blood cell count	8.8 x 10^3/mm3	4.5-11 x 10^3/mm3

**Figure 3:**
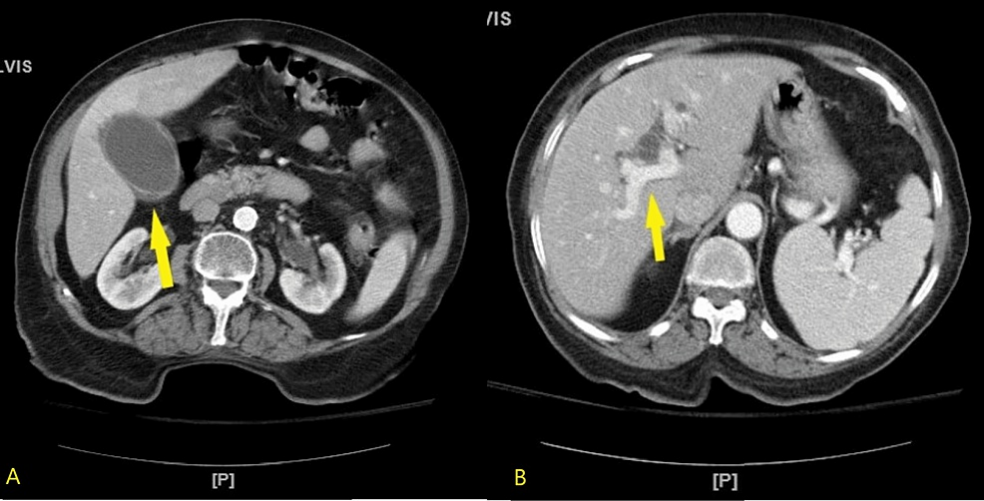
Computerized tomography of the abdomen demonstrating cholelithiasis with choledocholithiasis and acute cholecystitis Sagittal views of the abdomen and pelvis. The gallbladder is distended with wall thickening and pericholecystic fluid compatible with acute cholecystitis (yellow arrow, A). The biliary tree is dilated due to obstructive common bile duct calculi (yellow arrow, B).

A CT of the abdomen with contrast revealed cholelithiasis with choledocholithiasis and acute cholecystitis with distension of intrahepatic bile ducts and common bile duct (Figure 3).

**Figure 4:**
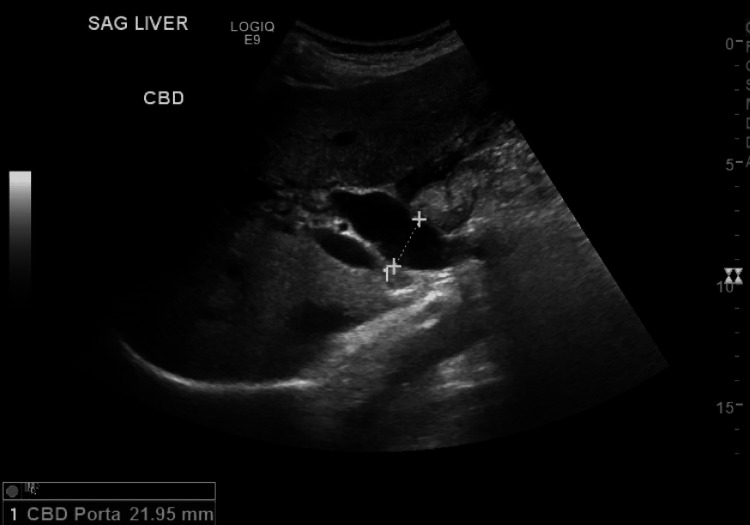
Ultrasound of the abdomen and pelvis revealing cholelithiasis and acute cholecystitis Sagittal view of the liver with measurements of the common bile duct, measuring approximately 22 mm.

Abdominal ultrasound revealed a distended, thickened, edematous gallbladder with pericholecystic fluid, sludge, and multiple stones within the lumen. The common bile duct measured 22 mm with a 12 mm obstructing stone (Figure 4).

She was diagnosed with acute cholecystitis, cholelithiasis, and choledocholithiasis. She was initially taken for ERCP however became severely bradycardic, and the procedure was aborted. A percutaneous cholecystostomy tube was placed for biliary decompression. The patient was deemed a poor surgical candidate, and she underwent a repeat ERCP. A localized, inflamed, and severely dilated biliary stricture was found in the lower third of the main bile duct. Partial removal of the stone was accomplished with a biliary sphincterotomy, and two stents were inserted into the main bile duct and ventral pancreatic duct, at which time purulent material was drained. Lithotripsy was successful in stone fragmentation, which was indicated as the stones were too large; however, removal was not complete. Three stones were visualized in the gallbladder upon subsequent ERCP, and a cholecystogastrostomy was created between the antrum and gallbladder using the AXIOS stent delivery system. Dilation with a 10mm anastomotic balloon was performed under direct visualization at the anastomosis. Electrohydraulic lithotripsy was successful, and the fragments of the gallstone were lavaged out of the gallbladder lumen. Prior to discharge, the percutaneous cholecystostomy was removed, and she was seen in the clinic approximately one month later with normalization of her liver enzymes and bilirubin. The AXIOS stent was removed approximately six weeks later. Upon further follow-up in four months' time, she underwent a magnetic resonance cholangiopancreatography which showed gallbladder decompression, no stones, and trace pericholecystic fluid. She is currently doing well without complications. 

## Discussion

Cholecystectomy remains the gold standard approach to the treatment of acute cholecystitis; however, many patients are poor surgical candidates [4]. Though percutaneous drainage is highly successful and minimally invasive, there are significant complications and risks associated with this approach, such as bleeding, bile leaks, and peritonitis, pneumothorax, perforation of the bowel, and catheter migration [4, 5]. Moreover, patients with ascites or on anticoagulation cannot undergo this procedure [4]. Recent advancements in EUS have led to endoscopic gallbladder decompression via the approach of transpupillary drainage or transmural stenting [4-6]. EUS-guided gallbladder decompression is minimally invasive with fewer complications, which is evidenced by numerous retrospective studies demonstrating high success rates, decreased leaks and/or stent migration, shorter hospital stays, and fewer unplanned admissions as compared to percutaneous drainage [4, 5, 7]. There is even an active prospective clinical trial directly comparing the AXIOS stent system to percutaneous gallbladder drainage in acute cholecystitis currently underway [4].

Though it has been well established over the last decade that endoscopic gallbladder decompression is a feasible, safe, and superior therapeutic modality for non-surgical candidates with acute cholecystitis, there remains the problem of patients with concomitant choledocholithiasis [6]. To our knowledge, only one other case report describes a patient who underwent both AXIOS stenting for biliary drainage as well as electrohydraulic lithotripsy for the treatment of calculous cholecystitis and choledocholithiasis [6]. Like the prior case report, both of our patients first had gallbladder decompression with the formation of a cholecystogastrostomy using the AXIOS stent system, and later, lithotripsy was performed in a subsequent procedure. In keeping with the ASGE SOP guidelines, sphincterotomy in conjunction with balloon dilation was performed on both patients, compared to sphincterotomy alone [2].

## Conclusions

Numerous clinical trials and retrospective studies have examined the efficacy and safety of endoscopic gallbladder drainage to treat acute cholecystitis; however, the role of concomitant endoscopic lithotripsy in non-surgical candidates remains unclear. We present two patients with calculous cholecystitis and choledocholithiasis who were successfully treated with EUS-guided decompression using the AXIOS stent, with subsequent EUS-guided lithotripsy. Future studies are warranted to determine the optimal approach to treating poor surgical candidates with large calculi and cholecystitis.
